# Sequencing Batch Integrated Fixed-Film Activated Sludge Membrane Process for Treatment of Tapioca Processing Wastewater

**DOI:** 10.3390/membranes11110875

**Published:** 2021-11-15

**Authors:** Nur Izzati Zainuddin, Muhammad Roil Bilad, Lisendra Marbelia, Wiratni Budhijanto, Nasrul Arahman, Afrilia Fahrina, Norazanita Shamsuddin, Zaki Ismail Zaki, Zeinhom M. El-Bahy, Asep Bayu Dani Nandiyanto, Poernomo Gunawan

**Affiliations:** 1 Department of Chemical Engineering, Universiti Teknologi PETRONAS, Seri Iskandar 32610, Malaysia; izzatizainuddin.work@gmail.com; 2 Faculty of Integrated Technologies, Universiti Brunei Darussalam, Bandar Seri Begawan BE1410, Brunei; norazanita.shamsudin@ubd.edu.bn; 3 Department of Chemical Engineering, Faculty of Engineering, Universitas Gadjah Mada, Jalan Grafika 2, Yogyakarta 55281, Indonesia; lisendra.m@ugm.ac.id (L.M.); wiratni@ugm.ac.id (W.B.); 4 Department of Chemical Engineering, Universitas Syiah Kuala, Banda Aceh 23111, Indonesia; afrilliafahrina26@gmail.com; 5 Magister Program of Environmental Management, Universitas Syiah Kuala, Banda Aceh 23111, Indonesia; 6 Department of Chemistry, College of Science, Taif University, P.O. Box 11099, Taif 21944, Saudi Arabia; zakimohamed@tu.edu.sa; 7 Department of Chemistry, Faculty of Science, Al-Azhar University, Nasr City, Cairo 11884, Egypt; zeinelbahy@azhar.edu.eg; 8 Department of Chemistry, Universitas Pendidikan Indonesia, Bandung 40154, Indonesia; nandiyanto@upi.edu; 9 School of Chemical & Biomedical Engineering, Nanyang Technological University, Singapore 639798, Singapore; pgunawan@ntu.edu.sg

**Keywords:** tapioca processing wastewater, gravity-driven membrane filtration, integrated fixed-film activated sludge, membrane filtration, novel bioreactor

## Abstract

Tapioca processing industries are very popular in the rural community to produce a variety of foods as the end products. Due to their small scales and scattered locations, they require robust modular systems to operate at low capacity with minimum supervision. This study explores the application of a novel sequencing batch-integrated fixed-film activated sludge membrane (SB-IFASM) process to treat tapioca processing wastewater for reuse purposes. The SB-IFASM employed a gravity-driven system and utilizes biofilm to enhance biodegradation without requiring membrane cleaning. The SB-IFASM utilizes the biofilm as a secondary biodegradation stage to enhance the permeate quality applicable for reuse. A lab-scale SB-IFASM was developed, preliminarily assessed, and used to treat synthetic tapioca processing industry wastewater. The results of short-term filtration tests showed the significant impact of hydrostatic pressure on membrane compaction and instant cake layer formation. Increasing the pressure from 2.2 to 10 kPa lowered the permeability of clean water and activated sludge from 720 to 425 and from 110 to 50 L/m^2^·h bar, respectively. The unsteady-state operation of the SB-IFASM showed the prominent role of the bio-cake in removing the organics reaching the permeate quality suitable for reuse. High COD removals of 63–98% demonstrated the prominence contribution of the biofilm in enhancing biological performance and ultimate COD removals of >93% make it very attractive for application in small-scale tapioca processing industries. However, the biological ecosystem was unstable, as shown by foaming that deteriorated permeability and was detrimental to the organic removal. Further developments are still required, particularly to address the biological stability and low permeability.

## 1. Introduction

Tapioca flour originated from the root of the cassava plant is used commercially as raw material in pharmaceuticals, textiles, cosmetics, food industries, and others [1]; and is very popular in tropical South-East Asian countries, where China dominates the imports. It has been reported that the annual demand has increased by 28.63% from 2006 until 2020 in Asia [2]. Small-scale tapioca processing industries are also common, especially in Asia, such as in Thailand and Indonesia. They operate traditionally, half mechanized, animal-energy, where the starch is extracted from cassava plants requiring a large quantity of water. The processes are mainly carried out semi-batch-wise in a one-day cycle. Therefore, the generated wastewater is also discharged intermittently, mainly in the morning (i.e., excessive water washing). Because the industries are minor and their locations are scattered, implementing the established wastewater treatment technology is untenable. Without appropriate wastewater treatment, the wastewater from cassava processing industries poses environmental challenges due to its adverse effects.

Without appropriate treatment, tapioca processing wastewater imposes a steep level of organic pollution, nitrogen, phosphorus, etc., due to its high organic content. It was reported that a high degree of organic matter is contained in a range of 13,000–17,700 mg/L in the wastewater [3,4]. Another study also reported the COD content of tapioca processing wastewater from a household processing unit of 6224–20,340 mg/L of COD. Moreover, it was reported that the COD of wastewater discharged from a small-scale tapioca processing is in a range of 3870–13,760 mg/L of COD [1]. Those reports unequivocally suggested that the high content of COD has been the main issue for cassava processing wastewater.

An anaerobic treatment method has been proposed to tackle the issue. The average removal of COD as high as 92.3–93.3% could be obtained under a long retention time of 22 days [1]. The process can primarily reduce the COD, but could not directly meet the industrial wastewater effluent discharge standards. The up-flow anaerobic sludge blanket (UASB) has also been shown to be effective in removing most COD, but still failed to achieve discharge standard as a standalone unit with poor stability in treating high levels of suspended solids [5]. The UASB system is also a bit complicated and requires an additional post-treatment system. Membrane bioreactors (MBRs), either aerobic or anaerobic, are newly established for wastewater treatment. They offer consistently high permeate quality at the expense of membrane fouling that inflates the energy input for the process [6]. Implementation of membrane filtration coupled with biological system thus seems promising for treating tapioca processing wastewater.

A concept of a gravity-driven membrane filtration system has been proposed as a new approach to naturally manage membrane fouling that otherwise is an Achilles’ heel of the traditional process [6]. It has proven to offer a sustained filtration process without membrane cleaning. It achieves a stable flux thanks to the mutual role of biofilm that is naturally a culprit causing membrane fouling [7]. The treated wastewater also experiences additional treatment by the biofilm leading to enhanced permeate quality [8,9,10]. Several reports emphasized that the transmembrane pressure (hereafter termed as hydrostatic pressure driven by gravity) affected membrane permeability and is thus considered as an essential parameter [11,12].

MBR employs bioactivated sludge for organic nutrients degradation and membrane filtration to polish the effluent. The use of high pressure driven by a pump in the MBR produces high flux yet promotes fouling. High flux drags the foulant, lets it accumulate, consolidates, and restricts the permeate flow. The membrane fouling requires an intensive cleaning using chemicals or mechanical disinfection. Biofilm forms on the membrane surface are the main culprit of membrane fouling and become a limitation for water filtration.

Applying low-pressure systems driven exclusively by gravity offers stable flux when treating surface water [7]. However, low flux can be compensated with a high membrane area [6]. The biofilm on the membrane surface is a secondary biodegradation process. Like in the MBR, activated sludge can be coupled with gravity-driven membrane filtration. However, instead of constantly removing the biofilm for membrane fouling control, it can be allowed to grow to emulate the traditional integrated fixed-film activated sludge system. The biofilm is formed on the membrane surface [13]. To our best knowledge, such a process has never been reported, let alone for treating the tapioca processing industry. Limited report is available on the fouling propensity of gravity driven membrane filtration treating feed with high solid content under long-term operation, which is the main issue addressed in this report.

The objective of this study is to explore the feasibility of a novel sequencing batch integrated fixed-film activated sludge membrane (SB-IFASM) process for the treatment of synthetic wastewater from tapioca processing industries. The novel process combines the traditional IFAS with a gravity-driven membrane process. The biofilm on the membrane surface is treated as the attached growth system in the traditional IFAS [13]. The application of the sequencing batch mode is to be adaptive to the nature of the cycle in the small-scale tapioca processing industries. The impact of hydrostatic pressure on the permeability of clean water and the activated sludge was first investigated, followed by the operation of an un-steady state lab-scale SB-IFASM to evaluate the filtration performance and COD removals.

## 2. Materials and Methods

### 2.1. Preparation of Activated Sludge

Twenty liters of activated sludge were collected at a nearby full-scale wastewater treatment plant to be used as inoculum. Two aerators were provided to ensure the dissolved oxygen (DO) level for the sludge liquor was maintained at >2 mg/L during the storage and the air flow rate was set at a constant rate of 1.5 L/min. This stage was treated as the acclimatization before being used as the seed sludge in the SB-IFASM systems. The sludge was fed every three days with 2 g of glucose (Sigma Aldrich, St. Louis, MO, USA), 3 g of potassium dihydrogen sulfate (KH_2_PO_4_·H_2_O, Supelco), 2 g of technical-grade urea (Petrokimia Gresik, East Java, Indonesia), and 30 g of tapioca flour (Yani maju, Yogyakarta, Indonesia), corresponded to feed COD of 1400–2000 mg/L. The formulation of the synthetic feed was based on the COD characteristics of local tapioca processing industries. The sludge conditions were recorded daily to observe any changes (i.e., bulking/foaming). The sludge was cultivated for a week before being used as the starting inoculum in the SB-IFASM system.

### 2.2. The SB-IFASM Set-Up

**Figure 1:**
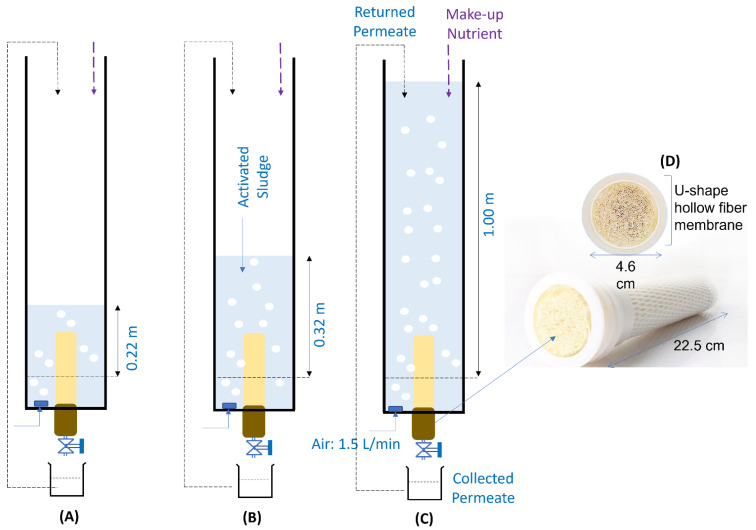
Illustration of the three reactors used for the experiment (**A**–**C**) and the picture of U-shape hollow fiber membrane installed in the reactors (**D**).

Figure 1 illustrates the SB-IFASM set-ups (A–C) and the dimension of the u-shape hollow fiber PAN ultrafiltration membranes (D) with a pore size of 0.01 µm. The PAN membrane was cleaned gently and wetted using ethanol solution before being installed in the SB-IFASM system. The hollow fiber membrane was soaked in a chlorine solution for two hours before being installed in the bioreactor. The PAN membrane was kept wet to prevent pore collapsing.

Three reactor set-ups made from acrylic materials were used in this study. The base was rectangular with both the width and length of 0.3 m, but with variable heights. Their effective height for liquid filling were 0.22, 0.32, and 1.00 m for reactor A, B, and C, respectively. Such heights corresponded to the hydrostatic pressures of 2.2, 3.2, and 10 kPa, respectively. Similar types of PAN membranes were placed in all SB-IFASM reactors. Each reactor was equipped with an aerator for mixing and supplying the DO for aerobic degradation of the organics at a constant rate of 1.5 L/min.

### 2.3. Short-Term Filtration Tests

The short-term filtration test was carried out using clean water to test the membrane’s clean water permeability. For the clean water permeability tests, the reactors were filled with clean water, then the permeate flux was recorded with 10 min of the time interval for an hour duration. The tests were run in triplicates. After the clean water tests, the set-ups were filled with the activated sludge (at different biomass concentrations) adjusted through dilution. The short-term filtration tests were then conducted with similar parameters. The permeate flux (J) and permeability (L) were calculated using Equations (1) and (2), respectively.

(1) $$J=\frac{V}{t\;A}\left(L/\left({h\;m}^{2}\right)\right)$$

(2) $$L=\frac{J}{\Delta P}\left(L/\left({h\;m}^{2}\;\mathrm{bar}\right)\right)$$

where V is the volume of permeate collected (*L*), A the membrane surface area (0.242 m^2^), t the time taken for the collected permeate (h), J is the permeate flux $\left(L/\left({h\;m}^{2}\right)\right)$, L is the permeability $\left(L/\left({h\;m}^{2}\;\mathrm{bar}\right)\right),$ and ΔP is the applied trans membrane pressure (bar).

### 2.4. The SB-IFASM Operation

The lab-scale SB-IFASM was operated under sequencing batch mode with an effective volume of the reactor of 32.1 L, in which the operation was split into two feeding modes over 43 days of operation. In feeding #1 ran from day 1 to day 23, the wastewater was fed once per day to the reactor to replace the volume loss as permeate. Some of the permeate was returned to the reactor to makeup water loss from evaporation (Figure 1). Due to an excessive foaming problem observed around days 20–23, the second feeding mode (Feeding #2) was implemented. The filtration ran continuously in feeding #2, but the permeate volume was replenished every two days. The samples were taken daily for the COD analysis and the total solids (TS) test. Meanwhile, the aerators were cleaned once a week to prevent clogging. In the SB-IFASM operation, no membrane cleaning was performed, and the system was run under unsteady-state operation. The synthetic feed was prepared by diluting sucrose (1 g), potassium dihydrogen sulfate (1.5 g), urea (1 g), and lastly tapioca flour (15 g) in 10 L of DI water. The feed had pH and turbidity of 7.5–7.8 and 105–107 NTU. Slightly alkaline pH was obtained due to addition of potassium dihydrogen sulfate. The pH was higher than typical acidic tapioca wastewater (pH-values: 4–6) and increasing of the pH benefited the biodegradation being optimum at pH around 8 [14].

### 2.5. Chemical Oxygen Demand Analysis

Liquid samples were injected into vials of 12 mL and centrifuged for 10 min. Then, the other vials were cleaned using H_2_SO_4_ solution using a pipette. The centrifuged solutions were then poured into the cleaned vials. After that, the heater was set to 150 °C. A total of 2.5 mL of distilled water was filled in a vial and labeled as a blank for the calibration. A total of 2.5 mL of centrifuged samples were put in each vial, followed by 1.5 mL of K_2_Cr_2_O_7_ and 3.5 mL of H_2_SO_4_. The prepared samples were shaken and wiped before being placed into the heater for 2 h. Lastly, the heated samples were cooled with distilled water before taking the COD readings. The COD measurements were conducted daily for the filtered mixed liquor, and the permeate samples. The COD removals were evaluated in term of overall removal (RT, %) and biofilm degradation (RB, %) as shown in Equations (3) and (4), where $COD_{F}$, $COD_{P}$, and $COD_{R}$ represent COD in the feed, permeate, and bioreactor, respectively.

(3) $$\;\mathrm{RT}=\frac{COD_{F}-COD_{P}}{COD_{F}}\;100\left(\%\right)$$

(4) $$\;\mathrm{RB}=\frac{COD_{R}-COD_{P}}{COD_{R}}\;100\left(\%\right)$$

### 2.6. Total Solid (TS)

The crucibles were first dried at 100 °C. Then, the cauldrons were weighed and recorded as the initial weight, $w_{i}$. The crucibles were then filled with 5 mL of sludge samples. The samples were heated for 3.5 h in an oven at 103 °C until dried. The dried crucibles were then weighed again and labeled as $w_{f}$. The total solids were calculated using Equation (5).

(5) $$TS\;\left(\frac{\mathrm{mg}}{L}\right)=\frac{w_{f}-w_{i}\times 1000}{Volume\;of\;the\;\mathrm{sample}}$$

## 3. Results and Discussion

### 3.1. Effect of Hydrostatic Pressure on the Clean Water and Activated Sludge Filtration

**Figure 2:**
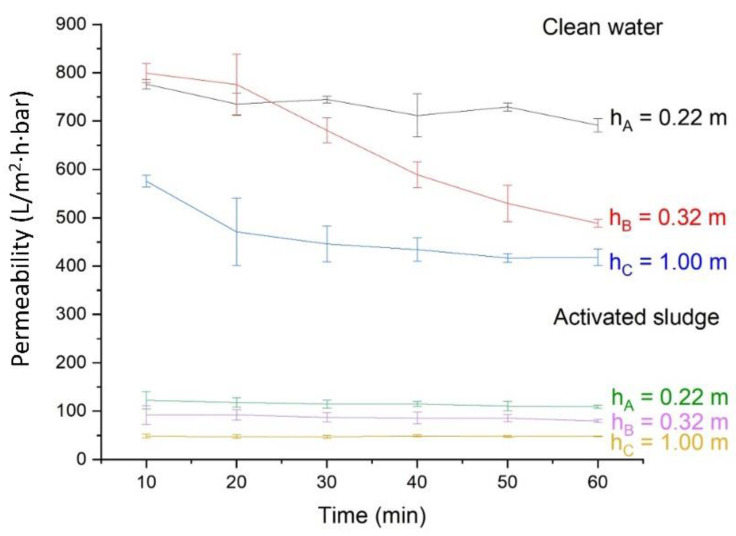
Clean water and activated sludge permeability function of hydrostatic pressure. The activated sludge filtration test was evaluated using TS of 2782 mg/L.

Figure 2 shows that at low transmembrane pressure, the hydrostatic pressure strongly affected the filtration of clean water and activated sludge. Three hydrostatic heights were tested, resulting in significant differences in the permeabilities, i.e., higher for the lower hydrostatic pressure. The highest permeability was shown by reactor A followed by reactor B and reactor C. The permeability readings fluctuated over time, primarily for reactor A, due to the fluctuation level of clean water due to air bubbling from activated sludge mixing, but a lesser degree of flux decline than others.

Figure 2 also shows the membrane permeability for the clean water, where reactor A had the highest value (of 720 L/m^2^·h bar), followed by reactor B (of 500 L/m^2^·h bar) and reactor C (of 426 L/m^2^·h bar). Reactor A with the lowest hydraulic pressure also showed the most fluctuated permeability readings. The three reactors experienced the same compaction tendencies (significant permeability decline over time) as they were made up from the same membranes and only differentiated by the applied pressure. The impact of hydrostatic pressure on the permeability observed in this study aligned with another low-pressure system reported earlier for filtration of detergent-containing wastewater [15].

The occurrence of membrane compaction can explain the significant impact of hydrostatic pressure on clean water permeability. It is generally agreed that clean water permeability is constant at any given pressure when the membrane reaches permanent compaction [16]. However, due to significantly lower applied pressure, the compaction phenomenon seems to be reversible, by judging from the reproducibility of the data, a consequence of the compaction. The effect of pressure at a low range (below 1 kPa) is very high on the clean water permeability, as reported elsewhere [17,18,19,20]. This finding indicates the need to develop a custom-made membrane that can resist compaction to maintain the hydraulic performance, as recently reported elsewhere [21,22].

The high degree of reproducibility of permeability data under multiple measurements suggested that the elasticity of the PAN polymer did not deform or compact easily under the applied pressure. The compaction phenomenon was highly reversible. The PAN polymer constricts when under pressure and expands back when the applied pressure is released. Previous studies have shown the increment of hydrodynamic resistance caused by the compaction of the membrane skin layer, which led to decrements of clean water and activated sludge permeabilities in line with an earlier report [19]. It is supported by the current findings within the range of the hydrostatic pressure. Despite showing higher permeability, the actual flux of the three reactors is almost similar.

### 3.2. Effect of Hydrostatic Pressure on the Activated Sludge Filtration

Filtration of activated sludge under different hydrostatic pressures somewhat followed a similar pattern with clean water filtration. The steady state permeabilities of the activated sludge filtration were much lower than the clean water, being 108.4, 79.8, and 50.0 L/m^2^·h bar with corresponding fluxes of 2.4, 2.9, and 5.1 L/m^2^·h for reactors A, B and C, respectively. The test was conducted using activated sludge with TS concentration of 2782 mg/L. The impact of the compaction phenomenon on permeability results is somewhat absurd. The lowest pressure led to the highest flux for the filtration of activated sludge, emphasizing the need for a custom-made membrane for a low-pressure system [21,22]. Therefore, the long-term filtration study in M-SBR-mode was carried out using reactor A at hydrostatic pressure of 2.2 kPa. The positive impact of low hydrostatic pressure on permeability allows the application of low hydrostatic pressure. It enabled the implementation of the system in a wastewater holding tank usually present in tapioca processing small-scale industries.

The cake layer fouling phenomenon explains the significant decline in the permeability of activated sludge. The average remaining permeability was merely 14% almost instantly at the beginning of filtration, from which the permeability remained stable. The cake layer seemed to develop immediately in the initial filtration stage, driven by the convective flow of the permeate. Under this condition, permeability decline can be attributed to the membrane compaction during clean water filtration. The rapid build-up of the cake layer became an additional filtration resistance. The extent of the cake layer build-up seemed to be affected by the hydrostatic pressure, possibly affecting the compaction of the cake layer. The absence of fouling control facilitated the development of the cake layer. As discussed later, the cake layer was expected to act as an additional biological agent in degrading the organics. A detailed study on the fouling mechanism under this condition shall be done in the future.

### 3.3. Effects of Activated Sludge Concentration on the Permeability

**Figure 3:**
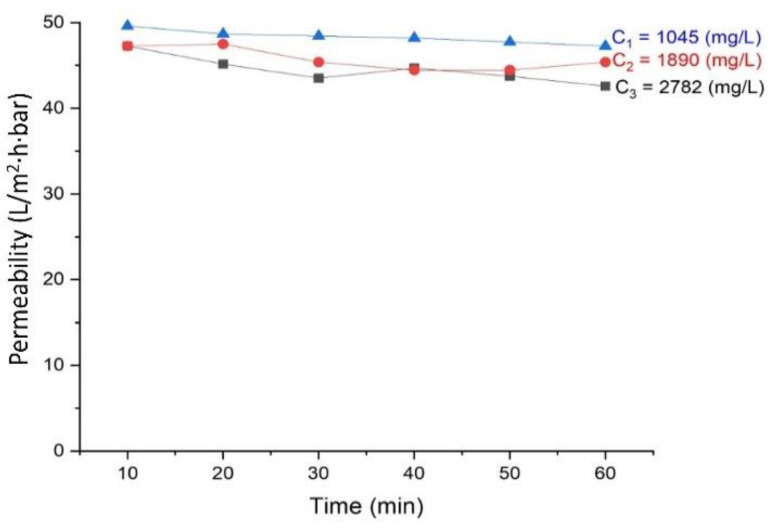
Effect of activated sludge concentration on permeability tested using reactor C.

Figure 3 shows that the high biomass concentration in the activated sludge has negatively affected the permeability. The finding is in line with the data in Figure 2, where a significant drop in permeability was observed when compared to the clean water permeability. The activated sludge concentrations of 2782, 1890, and 1045 mg/L resulted in permeability of 42.6, 45.4, and 47.3 L/m^2^.hr.bar, respectively. As expected, high feed concentration increased the filtration resistance as the foulant materials in the feed were gradually deposited on the membrane surface dragged by the permeate flow [15,23,24,25]. For the diluted activated sludge, less foulant was present, which thus allowed more flocs disaggregation [26]. This phenomenon suggests that the full-scale implementation of SB-IFASM would be restricted by the trade-off between activated sludge concentration and the flux.

The degree of fouling is more affected by the cake layer (thickness or compactness/porosity, rather than the extracellular polymeric substances typically occurs in the traditional MBRs) [27,28,29]. The membrane fouling also mainly occurred instantly during the initial stage of filtration and less so over the duration. The cake layer was immediately built up from the flocs dragged to the membrane surface by the convective flow of the permeate. Over time, the slight decline in permeability could be attributed to the consolidation of the flocs in the cake layer that enhanced cake compactness. High sludge concentration was more profound than the lower ones. The findings also indicated that hydrostatic pressure was more dominant than the sludge concentration when Figure 2 and Figure 3 are compared.

### 3.4. SB-IFASM Performances

#### 3.4.1. Filtration Performance

**Figure 4:**
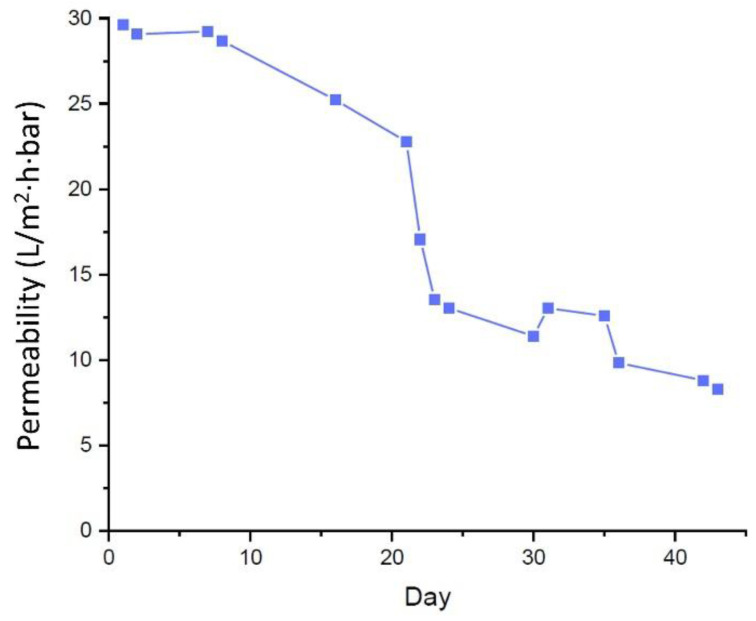
Permeability evaluation on membrane system in IFASM over long-term operation run using reactor A (of 0.22 m) at an initial TS concentration of 5.5 g/L.

Figure 4 shows that the permeability was almost stable during the first seven days even though the system was operated without any cleaning. After that, a steep decrement of permeability occurred, which can be attributed to the development of biofilm on the membrane surface [16]. Over time, after the cake formation was developed, the static cake layer allowed the microorganisms’ activity to evolve and stabilize for growth. They were originated from a network of biofilm linked by the exopolymeric particles. Consequently, the cake layer becomes denser, leading to higher hydraulic resistance for water permeation.

The permeability values shown in Figure 4 are also much lower than the ones in the traditional MBRs. The MBR permeabilities are typically higher as they maintain high permeability by applying chemical and physical cleaning. Hence, the SB-IFASM steady state permeability was lower than the typical conventional MBR on other gravity-driven systems [6,16]. The permeability was also influenced by the membrane characteristic and organic concentration [30]. The poor sludge quality was another unexpected factor, due to disrupted aeration [31]. Nonetheless, due to the absence of membrane fouling control in gravity-driven SB-IFASM, the operation turned out to be very simple with minimum energy input that is otherwise the most critical in the MBRs operations [32]. The adsoption was indeed occurred on the early stage of operation until the surface-active sites was saturated. Adsorption fouling is very common and only occurred in the first few mins/hours of the filtration [33,34]. The added tapioca flour was a factor that reduce the permeability since it was only partly (82%) biodegraded.

The long-term permeabilities were much lower than the short-term experiments (Figure 2 and Figure 3) since the activated sludge was thick and formed a cake layer. Also, the pressure applied was lower (of 0.02 bar). As organic loadings were abundant, the biofilm could not make enough contact time with the organics, resulted in fewer biological degradation [35].

The flux did not reach a steady value towards the end of 43 days of operation. The permeabilities were also lower than most gravity-driven systems treating other feeds [6]. The low permeability can be ascribed by the different nature of the feeds, most notably forming a thick biofilm layer in the SB-IFASM system. Despite the abundance of metazoan in adjusting biofilm offering flux stability [7], the permeability diminished over time. One important reason is sludge bulking (Section 3.4.2) around days 15–22. Even after the bulking issue was restored, the filtration permeability could not be restored. This finding demonstrates the potential issue of very low permeability in the SB-IFASM. Even though biofouling is utilized to enhance biological treatment, the membrane may still need cleaning after being used for an extended period. This approach has been suggested in maintaining the stability of other gravity-driven filtration systems [9,12]. Thus, the membrane clogging can be cleaned with a combination of physical and chemical cleaning for extensive foulant removals [30,31].

#### 3.4.2. Total Solid Mixing

**Figure 5:**
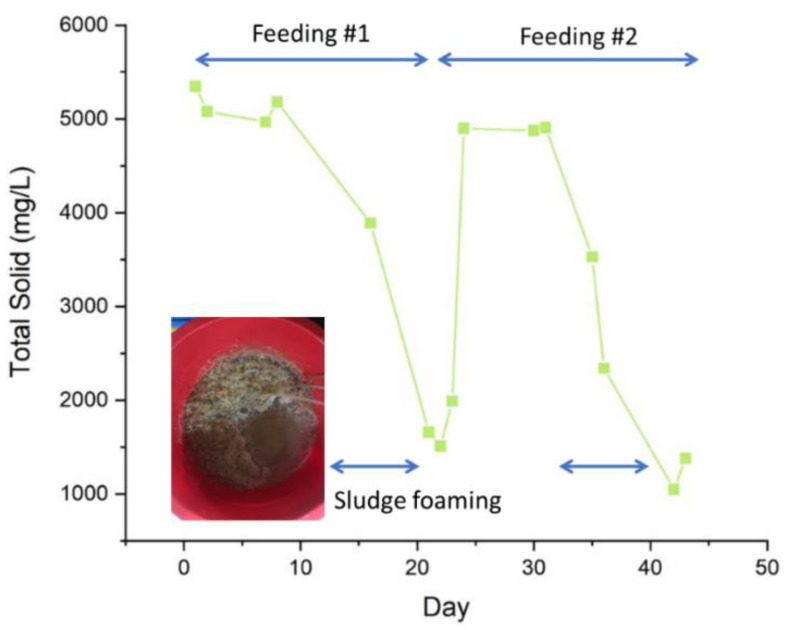
The profile of total solids over the SB-IFASM operation.

The declining trend of TS over time suggests the importance of mixing in an IFAS. The mixing applied in this study was not enough to sustain the stable suspension over time. A slow rate of aeration was applied to supply oxygen for aerobic degradation and to mix the activated sludge. The TS analysis was a crucial tool used to check the current biomass level of the sludge so the bacteria could be sustained throughout the experiment [36]. Figure 5 shows that the TS varied between 1000 mg/L and 5000 g/L. The first eight days show a stable TS concentration until it drops on day 13 until day 23. The rapid drop of the TS occurred due to partial sludge settling and due to sludge bulking. The inset of Figure 5 shows the image of sludge foaming. The foaming was caused by the abundance of organics due to the instant feeding mode [37,38]. Visually, it was observed that a significant amount of TS stuck on the bioreactor resulting in a drop of TS on day 16 to day 23 from 3890 to 1990 mg/L.

Because of the detrimental effect of the foaming on biological performance (Section 3.4.3) and filtration performance (Figure 4), the feeding rate was adjusted at day 23, which increased the TS on day 24 up to 4900 mg/L, and was stable until day 35 (with TS of 3528 mg/L). Then dwindled again until the end of the experiment (day 43). During this period (i.e., from day 35 until day 43), as the feeding was done from day 30, the readings displayed otherwise. As the operation progressed, the cake layer was denser, thicker, and hence needed more organic loadings. Hence, the portion was increased to feed the biofilm.

Concurrently, the sludge foaming also led to a significant drop in permeability (Figure 4). When considering the effect of sludge concentration (Figure 3), one can expect an increase in the permeability if the TS drops. However, a steep decline in permeability occurred. The finding highlights the importance of maintaining biological stability in the SB-IFASM. As recommended elsewhere, feeding management is an essential factor to be explored to manage the foaming issue [11,39].

#### 3.4.3. Chemical Oxygen Demand Removal

**Figure 6:**
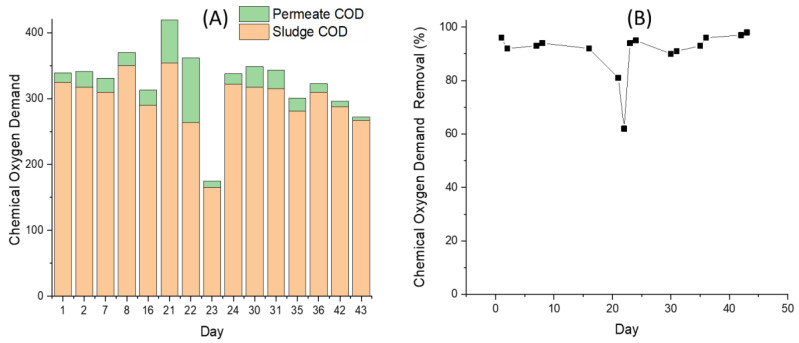
(**A**) Chemical oxygen demand permeate and mixed liquor and (**B**) the removal efficiencies showing the contribution of the biofilm in removing organics. Notice that the feed and the permeate COD values in (**A**) are stacked.

Figure 6 shows that the COD biofilm removals were consistently high at range of 63–98% evaluated using Equation (4). The removal efficiency was calculated by comparing the $COD_{p}$ of 5–98 mg/L and the filtered sludge ($COD_{R}$) of 165–354 mg/L. The difference between filtered sludge and permeate COD can be attributed to the biofilm activity on the membrane surface. A high degree of organic matter degradation was achieved thanks to the active role of biofilm. The consistently high COD removal rates by the biofilm were achieved, except on days 21 and 22 for 82 and 63%, respectively, during excessive foaming was observed that affected the biofilm and the cake layer [40,41]. In this first preliminary study, the effectiveness of the biofilm formed on the membrane surface in assisting biological performance was judge solely using COD removals. A more comprehensive analysis is indeed required when aiming for compliance with the wastewater discharge standard. The ultimate COD removals were >93% obtained by comparing the feed inlet COD of 1400–2000 mg/L with the final permeate COD of 5–98 mg/L evaluated using Equation (3).

After a short adjustment on the organic loading (Feeding #2), the biofilm rapidly stabilized and the COD removal increased again and slightly varied until day 43. The biofilm was strongly affected by the changes in the feed composition [42]. The alteration of the feeding frequency improved COD removal. The permeate COD then slightly fluctuated because of the sludge, biofilm dynamic, and biofilm conditions.

The high COD removal of >90% by the biofilm (RB) for the SB-IFASM demonstrated the enhanced role of the biofilm on the membrane surface in enhancing the biodegradation rate. The values were about 5% when compared to an UASB-reactor reported earlier [1], despite the simplicity in SB-IFASM operation. The operation of an SB-IFASM system is much simpler than an UASB-reactor. Application of UASB-reactor for tapioca wastewater requires a pre-treatment because the suspended solids in the tapioca processing wastewater may impair its reactor’s efficiency. In addition, pH adjustment—as applied in this study—might be required to allow application of the SB-IFASM. The results suggest that the SB-IFASM should be considered an attractive method for treating intermittently discharged tapioca processing wastewater in home-scale industries. Judging from the high removal of the COD, the permeate meets the discharge limit and has a high potential to be reused in some stages of tapioca processing industries (i.e., for washing purposes).

## 4. Conclusions

This study explores the feasibility of applying gravity-driven SB-IFASM operated under sequencing batch mode for treatment of tapioca industry using synthetic wastewater. Short-term filtration tests for clean water and activated sludge filtrations showed a significant impact of pressure compacting the membrane, thus lowering permeability. Increasing the pressures from 2.2 to 10 kPa lowered the clean water and sludge permeabilities from 720 to 425 and from 110 to 50 L/m^2^.hr.bar, respectively. The findings indicated the critical role of the pressure-promoted membrane compaction and rapid cake layer formation on the membrane surface without in situ membrane cleaning. The unsteady-state operation of the SB-IFASM under gravity-driven filtration and sequencing batch mode showed the prominent role of the bio-cake in removing the COD to reach a permeation quality suitable for reuse purposes. However, the activated sludge was found to be very unstable, as shown by foaming formation that deteriorates permeability, and also inappropriate mixing leads to settling of the sludge that further detriments the COD removal. Constantly high COD removals demonstrated the contribution of biofilm in enhancing biological performance (63–98%), made it attractive for application in small-scale tapioca processing industries. Further development to improve the SB-IFASM process is still required to address the biological stability and low filtration permeability.

## Data Availability

Not applicable.
